# Curiosities of Coal

**Published:** 1873-02

**Authors:** 


### CURIOSITIES OF COAL.

An average Atlantic steamer consumes fifty tons of coal in twenty-four hours. Five tons of coal will feed an ordinary grate in our dwellings during the entire season Hence the coal consumed on board a steamer in one day would last a small family, burning one fire, ten years.

If a load of coal is left out of doors, exposed to the weather until it is burned up in one grate, say a month, it loses one third of its heating quality.

If a ton of coal is dumped on the ground and left there, and another load is under a shed, the latter loses about twenty-five per cent. of its heating power; the former forty-seven per cent. Hence it is a great saving of coal to have it in a dry place, covered over, and on all sides.

The softer the coal, the more it loses, because the most volatile and valuable constituents undergo a slow combustion.

Coal must be kindled with wood, because each lump of coal must have enough caloric or heat sent into it to raise it to a certain temperature before it is capable of catching, that is, turning red and coming to a flame. But it requires only half the amount of heat to bring a lump of coal weighing one pound to the burning point, as if it weighed two pounds; hence the smaller the bits of coal, the sooner they will kindle, and the less wood will it require. Bits of coal not larger than a filbert will catch to burning in half the time required for coal as large as a hen's egg.

If you concentrate the wood and put the bits of coal around it closely, it will catch very soon. But if you will go into the kitchen and watch the cook while she builds up the fire, you will see that she goes about it in the most wasteful way possible; the wood is scattered over the whole bottom of the grate, instead of at one side or the middle.

It puts one in the best possible humor, to go into the breakfast room of a cold winter's morning, and find the fire cheerfully blazing, and the whole apartment delightfully warm. But suppose you dress without a fire, the thermometer hugging zero outside, and you come down all doubled and drawn up and wizened with the cold, to look at a black mass in the grate that "won't burn anyhow," and the more you poke it the more it won't kindle: you are sadly "put out" about it, to say the least. If you are a gentleman or lady, you say nothing, and make the best of it; but as there are few persons of that character, the general course of affairs is a continuous series of scowls and frowns and questions and cross-questions to the servants, and short, angry answers to everybody else, with the result, but too often, that the breakfast is eaten in sad and solemn silence; the day has begun with a cloud, and is spoiled, not merely for a grumpy husband or a nervous, fretful, complaining wife, but for the children also; the servants catch the infection, become cross-grained, and do nothing cheerfully or well, and all because the servants did not know how to kindle a coal fire, — nor master nor mistress either.

